# CryoEM Reveals the Complexity and Diversity of ATP Synthases

**DOI:** 10.3389/fmicb.2022.864006

**Published:** 2022-06-16

**Authors:** Gautier M. Courbon, John L. Rubinstein

**Affiliations:** ^1^Molecular Medicine Program, The Hospital for Sick Children, Toronto, ON, Canada; ^2^Department of Medical Biophysics, The University of Toronto, Toronto, ON, Canada; ^3^Department of Biochemistry, The University of Toronto, Toronto, ON, Canada

**Keywords:** ATP synthase, cryoEM, protein, structure, membrane, bioenergetics

## Abstract

During respiration, adenosine triphosphate (ATP) synthases harness the electrochemical proton motive force (PMF) generated by the electron transport chain (ETC) to synthesize ATP. These macromolecular machines operate by a remarkable rotary catalytic mechanism that couples transmembrane proton translocation to rotation of a rotor subcomplex, and rotation to ATP synthesis. Initially, x-ray crystallography, nuclear magnetic resonance (NMR) spectroscopy, and cross-linking were the only ways to gain insights into the three-dimensional (3D) structures of ATP synthases and, in particular, provided ground-breaking insights into the soluble parts of the complex that explained the catalytic mechanism by which rotation is coupled to ATP synthesis. In contrast, early electron microscopy was limited to studying the overall shape of the assembly. However, advances in electron cryomicroscopy (cryoEM) have allowed determination of high-resolution structures, including the membrane regions of ATP synthases. These studies revealed the high-resolution structures of the remaining ATP synthase subunits and showed how these subunits work together in the intact macromolecular machine. CryoEM continues to uncover the diversity of ATP synthase structures across species and has begun to show how ATP synthases can be targeted by therapies to treat human diseases.

## Introduction

In eukaryotes and aerobic bacteria, the synthesis of most of the cell's adenosine triphosphate (ATP) is accomplished by the combined activities of the electron transport chain (ETC) and ATP synthase. Reduced nicotinamide adenine dinucleotide (NADH) produced by glycolysis, fatty acid oxidation, and the Krebs cycle, as well as succinate from the Krebs cycle, are oxidized by the integral membrane protein complexes of the ETC. Electrons pass between the complexes *via* the intermediate electron carriers quinone and cytochrome *c* before ultimately being used to reduce oxygen to water. Within some of the ETC complexes, these redox reactions are coupled to proton translocation across the membrane, either from the mitochondrial matrix to the mitochondrial intermembrane space in eukaryotes or from the cytoplasm to the periplasm or extracellular environment in bacteria. This activity establishes an electric field (Δψ) and an ion gradient (ΔpH) that result in a proton motive force (PMF) across the membrane. In the final step of oxidative phosphorylation, ATP synthases harness the PMF to synthesize ATP from adenosine diphosphate (ADP) and inorganic phosphate (Pi). The hydrolysis of this ATP is then used by a multitude of enzymes as an energy source to catalyze energetically unfavorable reactions. Significant structural knowledge of ATP synthase subunits and subcomplexes was obtained through pioneering efforts with x-ray crystallography (Abrahams et al., 1994; Stock et al., 1999; Dickson et al., 2006). This work provided insight mostly, though not exclusively, into the structure of the soluble region of the enzyme. Early electron cryomicroscopy (cryoEM) was limited to fitting crystal structures together, like pieces of a puzzle, to form a “mosaic model” of intact ATP synthases (Rubinstein et al., 2003; Lau et al., 2008; Baker et al., 2012). More recently, as described in this review, cryoEM has provided high-resolution structural knowledge for the missing pieces of the puzzle and offered a detailed picture of how intact ATP synthases work. These studies continue to reveal the complexity and diversity of ATP synthases across species.

## Overall Structures of ATP Synthases

**Figure 1:**
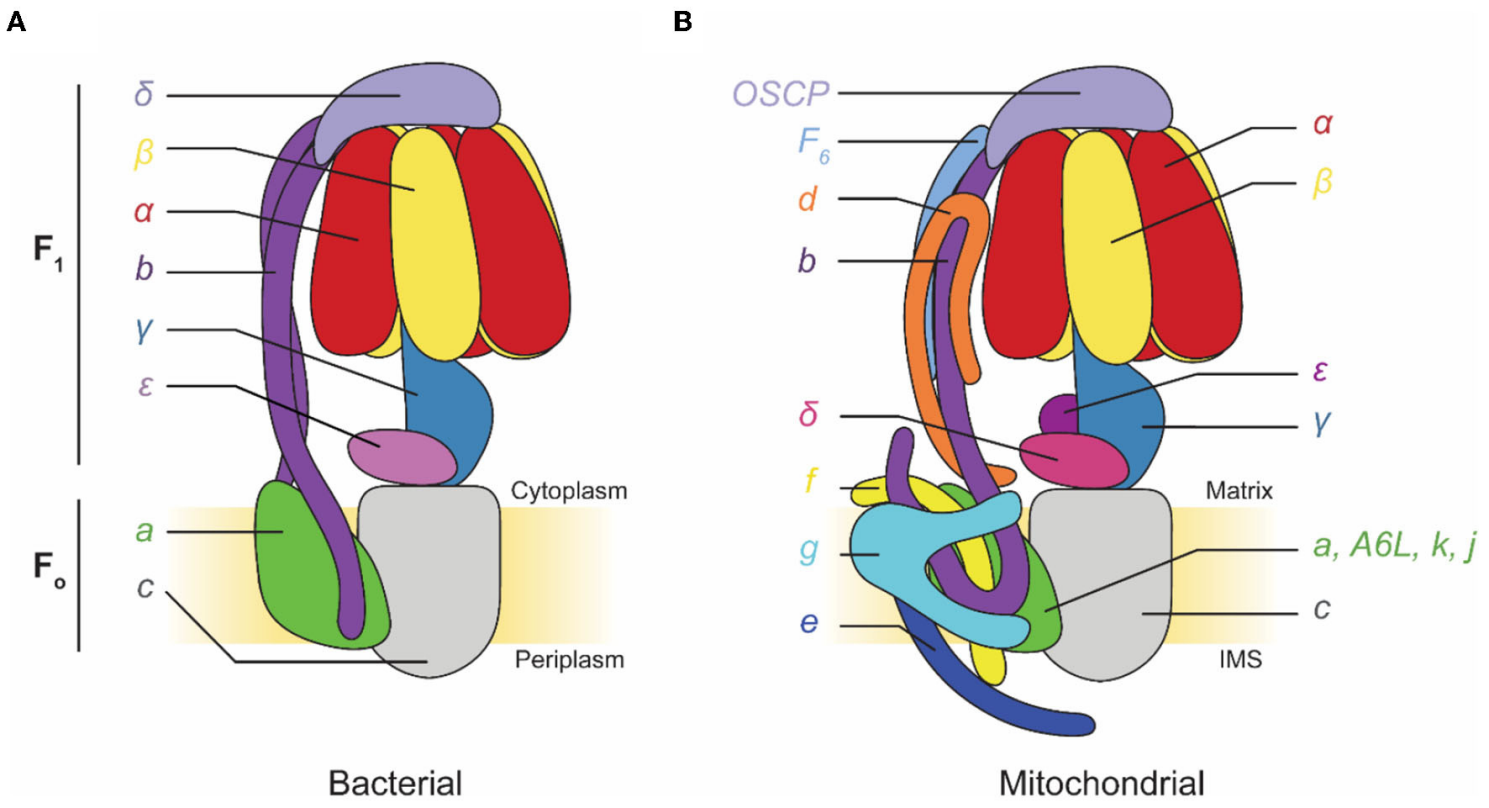
Subunit composition of commonly studied adenosine triphosphate (ATP) synthases. **(A)** Cartoon of a bacterial ATP synthase. Based on Guo et al. (2019). **(B)** Cartoon of a mammalian mitochondrial ATP synthase. IMS, intermembrane space.

F-type ATP synthases are multi-subunit complexes consisting of a catalytic F_1_ region and a membrane-embedded F_O_ region (Figure 1). Electron microscopy first detected the F_1_ regions of ATP synthases protruding from mitochondrial membranes like lollipops in images of specimens prepared with heavy metal salt stain (Fernández-Morán, 1962; Kagawa and Racker, 1966a,b). The F_1_ region catalyzes ATP synthesis but can also hydrolyze ATP under some conditions. F_1_ is composed of three pairs of α and β subunits that form a ring around a central stalk consisting of subunits γ, δ, and ε in eukaryotes, or subunits γ and ε (a δ homolog) in prokaryotes (Walker et al., 1982, 1985; Abrahams et al., 1994; Stock et al., 1999; Gibbons et al., 2000). ATP synthesis or hydrolysis in the F_1_ region is coupled to rotation of the central stalk within the α_3_β_3_ hexamer. This central stalk is firmly attached to a ring of membrane-embedded c subunits that form part of the F_O_ region (Stock et al., 1999; Jiang et al., 2001; Seelert et al., 2003). In bacteria, the remainder of the F_O_ region consists of subunit a and a transmembrane α helix from each of two b subunits. Subunit a functions with the c ring to allow proton translocation while the two b subunits form a peripheral stalk that prevents F_1_ and F_O_ from rotating relative to each other when the central stalk and c ring turn. In chloroplasts and some bacteria, this peripheral stalk is formed by a bb′ heterodimer rather than a b_2_ homodimer. In mitochondrial ATP synthases, such as the enzyme from *Saccharomyces cerevisiae*, F_O_ includes subunits a, e, f, g, i/j (sometimes called 6.8PL in mammals), k (DAPIT in mammals), 8 (A6L in mammals), and a transmembrane portion of subunit b. The yeast peripheral stalk is formed from subunits b, d, h (known as F_6_ in mammals), and the oligomycin sensitivity conferral protein (OSCP) (Liu et al., 2015; He et al., 2018). The number of c subunits in the c ring varies among species, with bacteria possessing between 9 (Preiss et al., 2015) and 15 (Pogoryelov et al., 2009) copies, yeast having 10 c subunits (Stock et al., 1999), and all animals proposed to have eight c subunits (Watt et al., 2010). Proton translocation through the interface of subunits a and c induces c ring rotation, which rotates subunit γ within the α_3_β_3_ hexamer and drives ATP synthesis.

## Simultaneous Development of Electron Microscopy and Structural Models of ATP Synthases

Developments in electron microscopy have contributed increasingly to the understanding of the structure and function of ATP synthases. After realizing that coupling of F_1_ and F_O_ activities requires a peripheral stalk structure (Engelbrecht and Junge, 1997), electron microscopy of negatively stained ATP synthase particles from a variety of species revealed a faint feature corresponding to the stalk in two-dimensional (2D) class average images (Böttcher et al., 1998, 2000; Wilkens and Capaldi, 1998; Karrasch and Walker, 1999). This structure became clearly visible in three-dimensional (3D) maps with the advent of single-particle cryoEM methods for membrane proteins, which were initially limited to 20–30 Å resolution (Rubinstein et al., 2003; Lau et al., 2008). These early cryoEM structures relied on the availability of relatively stable cryospecimen holders for microscopes and highly coherent field emission electron sources, which are required to provide contrast in defocused images of protein complexes. However, the detective quantum efficiency of the photographic film used with microscopes remained relatively low, limiting the resolution of 3D reconstructions. Despite this limitation, cryoEM revealed density for the F_1_ region, the peripheral stalk, the c ring, and a large region of density corresponding to the additional membrane protein subunits of the mitochondrial ATP synthase, most of which had unknown functions at the time. Perhaps most importantly, the structure of subunit a, which works together with the c ring to couple proton translocation to rotation, remained unknown. Advances in electron tomography revealed the 3D arrangement of mitochondrial ATP synthases in dimer ribbons (Strauss et al., 2008; Davies et al., 2011) that had previously been observed in freeze-etch electron microscopy (Allen et al., 1989). Gradual improvements in specimen preparation and image analysis allowed cryoEM of ATP synthases and related complexes at 10–20 Å resolution (Baker et al., 2012; Benlekbir et al., 2012; Lau and Rubinstein, 2012). However, this resolution still fell short of the 7–8 Å resolution needed to reliably detect α-helices in structures or the better than 4 Å resolution needed to detect amino acid side chains.

The development of direct detector device (DDD) cameras for electron microscopes led to a sudden increase in the resolution attainable by cryoEM (McMullan et al., 2016). This technology improved the resolution to 6–8 Å, which revealed highly tilted α-helices from the a subunit in contact with the c ring in both ATP synthases (Allegretti et al., 2015; Zhou et al., 2015; Sobti et al., 2016) and the related proton-pumping eukaryotic vacuolar-type (V-type) ATPases (Zhao et al., 2015). This progress coincided with solution of a crystal structure of a bacterial ATP synthase at comparable resolution (Morales-Rios et al., 2015).

## Dynamics of ATP Synthases

**Figure 2:**
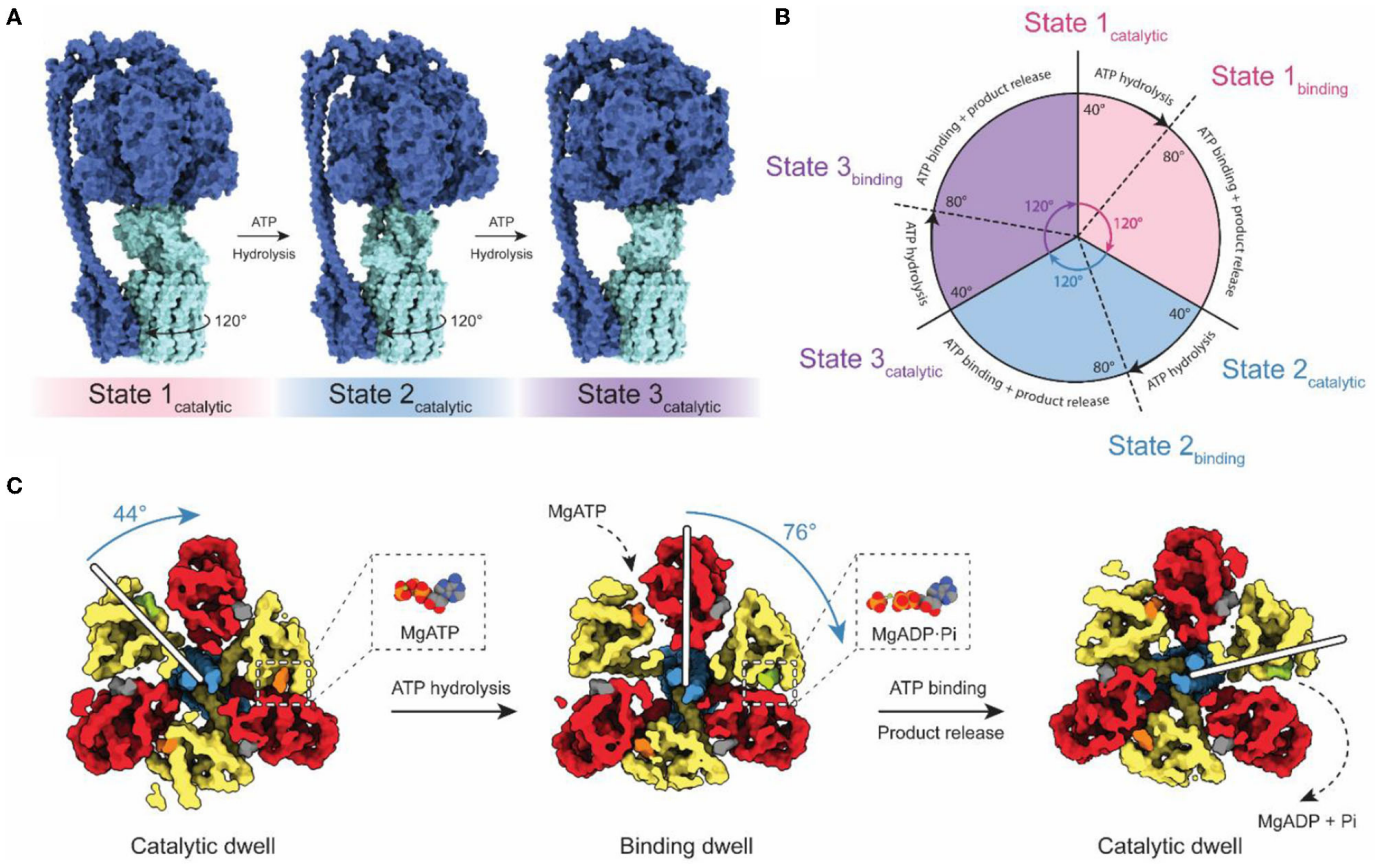
Rotary catalytic mechanism of ATP synthase. **(A)** Atomic model of *Bacillus* PS3 ATP synthase showing the three main rotational states (PDB: 6N2Y, 6N30, and 6N2Z) (Guo et al., 2019). The light blue parts of the complex rotate relative to the dark blue parts. The direction of rotation during ATP hydrolysis is indicated. **(B)** Diagram of the catalytic cycle of the F_1_ region during ATP hydrolysis. **(C)** Structures of the *Bacillus* PS3 F_1_ complex catalytic substeps (PDB: 7L1R and 7L1Q) (Sobti et al., 2021). White bars represent the angle of the γ subunit. ATP, ADP, and non-catalytic nucleotides are colored orange, green, and dark gray, respectively. Based on Sobti et al. (2021).

Although falling short of the 3–4 Å resolution needed to build atomic models, cryoEM with both field emission sources and DDD cameras gave rise to the study of ATP synthase dynamics by cryoEM (Figure 2). Three catalytic nucleotide-binding sites in the F_1_ region are located at the interface of each αβ pair, primarily within the β subunits (Abrahams et al., 1994). These catalytic sites were termed as β_TP_ (“ATP-bound”), β_DP_ (“ADP-bound”), and β_E_ (“Empty”), based on their nucleotide content in the first crystal structure of the F_1_ region. The structure of the bovine F_1_ region supported the earlier prediction of a rotary catalytic mechanism in which each nucleotide-binding site of ATP synthase cycles between the three different catalytic states *via* rotation of the central stalk relative to the α_3_β_3_ ring (Boyer, 1997). This rotation was observed experimentally by fluorescence microscopy using fluorescent actin filaments attached to the γ subunits of immobilized F_1_ complexes (Noji et al., 1997). Fluorescence studies also revealed that the ATPase cycle can be divided into three distinct 120° steps, which is consistent with the pseudo 3-fold symmetry of the F_1_ catalytic region (Yasuda et al., 1998).

The coexistence of these conformations in purified enzyme preparations complicates structural analysis by cryoEM, but also allows the possibility of direct visualization of the rotary cycle at a structural level. Methods to separate protein conformations that exist simultaneously in solution had been applied previously to ribosomes, viruses, and DNA helicases (Valle et al., 2002; Yang et al., 2002; Heymann et al., 2003; Gao et al., 2004; Scheres et al., 2007) but DDDs provided the signal-to-noise ratios in images needed to do this computational separation for ATP synthases and V-ATPases (Zhao et al., 2015; Zhou et al., 2015). The structures of rotational states show with striking clarity how rotor rotation within the α_3_β_3_ hexamer drives the conformational transition between each of the three 120° catalytic steps (Figure 2A; Zhao et al., 2015; Zhou et al., 2015; Sobti et al., 2016; Hahn et al., 2018; Guo et al., 2019). Movies made from interpolation between rotational states suggest remarkable flexibility in the enzyme, allowing it to twist and deform to accommodate the symmetry mismatch between the 3-fold symmetric F_1_ region and the higher symmetry in the c ring within the F_O_ region.

High-speed fluorescence microscopy subsequently detected the existence of F_1_ substeps during the catalytic cycle, dividing each 120° step into a short ~40° step and long ~80° step (Figures 2B,C; Yasuda et al., 2001; Bilyard et al., 2013; Martin et al., 2014). With limiting amounts of ATP, the dwell preceding the ~80° step becomes longer, while the dwell preceding the ~40° step remains the same (Yasuda et al., 2001). This observation suggests that the ~80° step is induced by ATP binding (the “binding dwell”) while the ~40° step is independent of ATP concentration and coincides with catalysis (the “catalytic dwell”) (Yasuda et al., 2001). While the catalytic dwell conformation was detected in the earliest crystallographic structures, structural evidence for the F_1_ binding dwell was first determined two decades later (Sobti et al., 2021). These structures confirmed the existence of a structurally distinct catalytic dwell and binding dwell, with a 44° rotation of the rotor during the ATP hydrolysis stroke and a 76° rotation during ATP binding at one site and product release from another site. The substep composition of catalytic cycles can differ between organisms (Zarco-Zavala et al., 2020). Notably, single-molecule and x-ray crystallographic data have shown that mammalian ATP synthases possess an additional substep interpreted as a pre-phosphate release state (Suzuki et al., 2014; Bason et al., 2015). However, this state has not been observed in bacterial or yeast ATP synthases by either single-molecule experiments or structural studies.

## High-Resolution Structures of the F_O_ Region Obtained by Chemical or Computational Isolation From F_1_

While providing movies of the conformational changes of ATP synthases during rotary catalysis, cryoEM maps at 6–8 Å resolution are not sufficient to build atomic models of subunits in the F_O_ region. These maps could be combined with evolutionary covariance (Marks et al., 2011), a technique central to the success of recent protein structure prediction methods (Senior et al., 2020), to determine the a subunit fold (Zhou et al., 2015; Schep et al., 2016). However, they lacked the high-resolution detail needed to determine amino acid side chain orientations, which cryoEM with a DDD can provide for other proteins (Cao et al., 2013; Bai et al., 2015a). One hypothesis for the limited resolution in ATP synthase structures was that conformational heterogeneity of the enzyme blurs the structure even following computational separation of the different rotary states. In support of this hypothesis, structure determination of the membrane-embedded V_O_ region of V-ATPase following physiological separation of V_1_ and V_O_ reached a resolution of 3.9 Å, sufficient to build an atomic model of that complex (Mazhab-Jafari et al., 2016). Based on this idea, a first high-resolution structure of the F_O_ region was determined by chemically separating F_1_ from F_O_ with sodium bromide before structure determination. This approach allowed the construction of an atomic model for the dimeric yeast F_O_ complex more than 20 years after the first high-resolution F_1_ structure (Guo et al., 2017).

**Figure 3:**
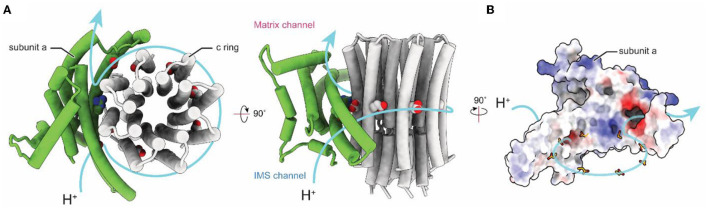
Proton translocation in ATP synthase. **(A)** Proton path (blue arrow) through the interface between the a and c subunits of the F_O_ region of bovine ATP synthase (PDB: 6ZPO) (Spikes et al., 2020). The conserved arginine in subunit a and acidic residues in the c ring are shown as space filling models. **(B)** Electrostatic surface of bovine subunit a, with positively and negatively charged surfaces colored blue and red, respectively.

Computational separation of regions of a protein structure that move relative to each other offers an alternative method for gaining high-resolution insights into flexible proteins (Bai et al., 2015b). This method allows a computational equivalent of chemical separation, in which the structures of different parts of the enzyme can be determined at high resolution independently and then fit together to generate an overall composite high-resolution map. These techniques include separation of conformations with maximum-likelihood classification (Scheres et al., 2007), selection of an area within a 3D map for high-resolution refinement, and signal subtraction to eliminate the contribution of regions outside the mask during particle image alignment. The combination of these evolving computational methods for 3D classification and focused refinement in software packages such as Relion (Scheres, 2012), Frealign/cisTEM (Grigorieff, 2016), and cryoSPARC (Punjani et al., 2017) allowed for high-resolution insights into the F_O_ regions of chloroplasts (Hahn et al., 2018), bacteria (Guo et al., 2019), algae (Murphy et al., 2019), mammalian mitochondria (Spikes et al., 2020), and protozoan mitochondria (Mühleip et al., 2019, 2021; Flygaard et al., 2020). High-resolution structures of F_O_ regions revealed a similar topology of residues important for proton translocation in both ATP synthase and V-type ATPase a subunits (Mazhab-Jafari et al., 2016; Schep et al., 2016). The structures show two offset proton half-channels in subunit a that allow the passage of protons to and from the c ring (Figure 3; Vik and Antonio, 1994; Junge et al., 1997). Protons are carried between the half-channels by conserved acidic residues in the c subunits, with rotation of the ring taking the proton from one half-channel, through the hydrophobic environment of the lipid bilayer, and to the second half-channel before its release. A conserved arginine in subunit a produces a positive charge on the surface of the a subunit where it contacts the c ring. This arginine residue prevents a short circuit in which protons can pass from one half-channel to the other without inducing ring rotation.

## CryoEM Reveals the Diversity of ATP Synthases

**Figure 4:**
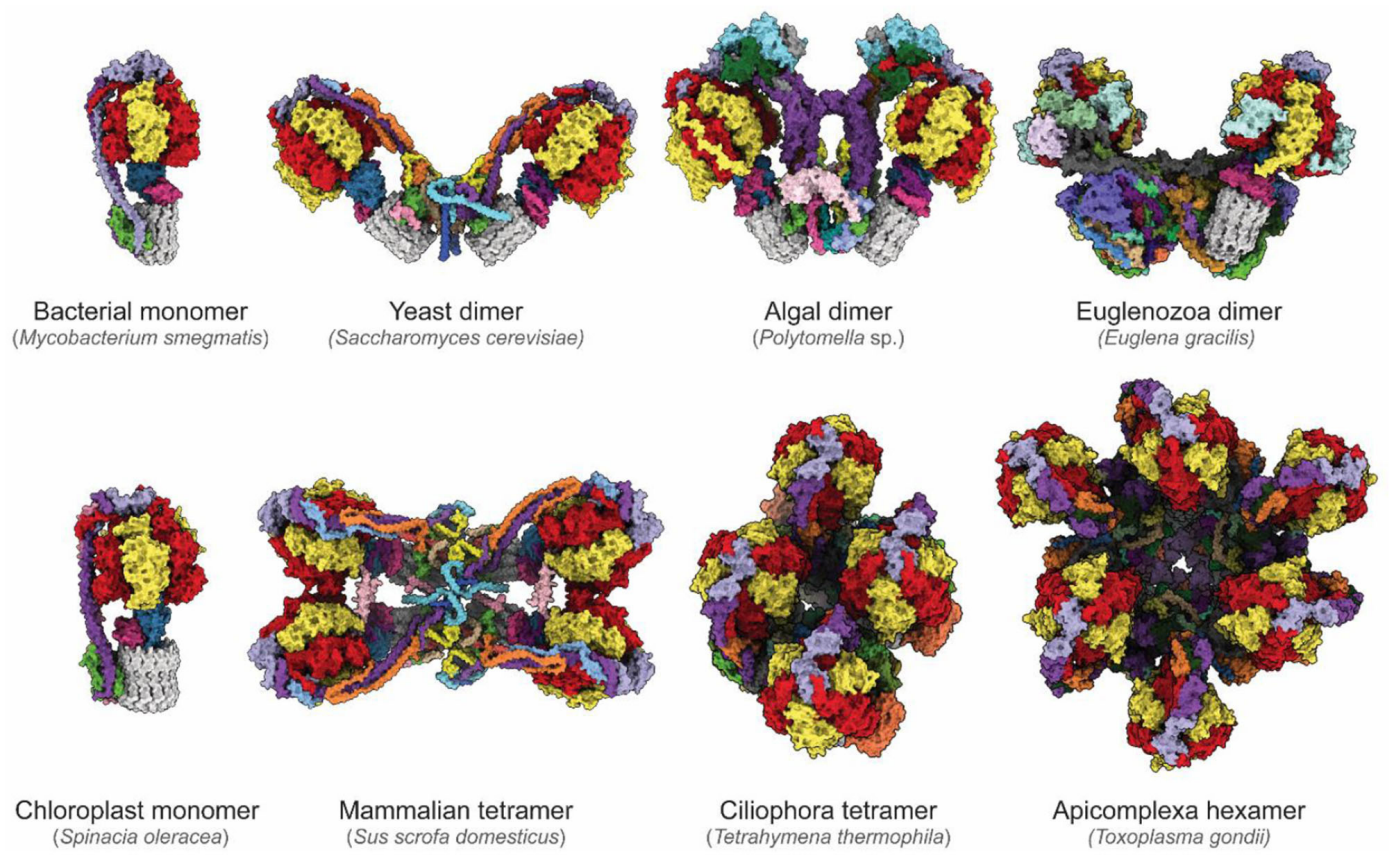
Diversity of ATP synthases. Atomic models of ATP synthases from diverse species. Homologs of subunits α, β, γ, δ, ε, and c are colored red, yellow, blue, pink, purple, and gray, respectively. From the left to right, top to bottom: *Mycobacterium smegmatis* (PDB: 7JG5) (Guo et al., 2021), *Saccharomyces cerevisiae* dimer (PDB: 6B8H) (Guo et al., 2017), *Polymotella* sp. dimer (PDB: 6RD4) (Murphy et al., 2019), *Euglena gracilis* dimer (PDB: 6TDU) (Mühleip et al., 2019), *Spinacia oleracea* (PDB: 6FKF) (Hahn et al., 2018), *Sus scofa domesticus* tetramer (PDB: 6J5K, 6ZNA) (Gu et al., 2019; Spikes et al., 2020), *Tetrahymena thermophila* tetramer (PDB: 6YNZ) (Flygaard et al., 2020), and *Toxoplasma gondii* hexamer (PDB: 6TML) (Mühleip et al., 2021).

The newfound ability to routinely determine high-resolution structures of ATP synthases has revealed remarkable diversity in these essential enzymes (Figure 4). While bacterial and chloroplast ATP synthases are monomeric, the mitochondrial enzyme forms higher-order oligomers. One area in which diversity between species occurs is the way in which monomers of ATP synthases assemble into larger dimers and dimer ribbons in mitochondria. CryoEM of an algal ATP synthase from *Polytomella* sp. (Klusch et al., 2017; Murphy et al., 2019) revealed a large and rigid peripheral stalk structure with numerous algae-specific subunits that results in the complex purifying in dimeric form even with relatively harsh detergents (Dudkina et al., 2005). In contrast, in yeast dimerization is mediated by fragile interactions between subunits e, k, i/j and a (Guo et al., 2017). In addition to determining the structures and identities of corresponding subunits in yeast and mammalian F_O_ regions (Hahn et al., 2016; Vinothkumar et al., 2016; Guo et al., 2017; Srivastava et al., 2018; Spikes et al., 2020), cryoEM of mammalian ATP synthases has allowed definition of the structure of higher-order contacts in dimer ribbons. A structure of a mammalian ATP synthase tetramer from porcine heart (Gu et al., 2019) was interpreted with insights from structures of a mammalian ATP synthase monomer isolated from ovine heart (Pinke et al., 2020) and of a mammalian ATP synthase dimer from bovine heart (Spikes et al., 2020). This analysis revealed that two inhibitory factor 1 (IF1) proteins link two dimers, with the dimer–dimer interaction stabilized by the N-terminal portions of subunits k and g above the membrane, and subunit e within the membrane. These mammalian ATP synthase structures also show that the C-terminal region of subunit e points toward the c ring within the monomer (Gu et al., 2019; Pinke et al., 2020; Spikes et al., 2020).

The diversity of enzyme architecture is perhaps most strikingly illustrated by recent structures of mitochondrial ATP synthases from *Euglena gracilis* of the phylum Euglenozoa (Mühleip et al., 2019), the intracellular parasitic protozoan *Toxoplasma gondii* of the phylum Apicomplexa (Mühleip et al., 2021), and the ciliated protozoan *Tetrahymena thermophila* of the phylum Ciliophora (Flygaard et al., 2020). These structures reveal highly elaborated enzymes with numerous additional subunits that influence monomer–monomer interactions, which in turn can lead to different oligomerization and cristae structures. These enzymes produce remarkable arrangements ranging from stable dimers that induce discoid cristae in Euglenozoa (Mühleip et al., 2019), tetramers that assemble into helical rows to induce the formation of tubular cristae in Ciliophora (Mühleip et al., 2016; Flygaard et al., 2020), and even hexamers that assemble into larger pentagonal pyramids to induce bulb-shaped cristae in Apicomplexa (Mühleip et al., 2021). Although in each case additional ATP synthase subunits and modifications of core subunits reveal *how* the enzyme shapes cristae, it remains unclear *why* cristae adopt these different shapes, and what physiological advantage the different cristae morphologies confer. Consequently, the specific roles of these additional subunits and of the diversity of mitochondrial membrane morphologies are a rich area of investigation.

A second area of diversity in the ATP synthase structure relates to how ATP hydrolysis is inhibited in different species in the absence of a PMF. In mammals and yeast, this role is filled by IF1, which binds and inhibits the enzyme upon PMF collapse (Cabezón et al., 2003). The structure of the chloroplast ATP synthase (Hahn et al., 2018) showed that the γ subunit contains a double-hairpin that acts as a redox sensor, where oxidation of a disulfide bond can prevent ATP hydrolysis. In many bacteria, the ε subunit can insert into the α_3_β_3_ hexamer to block ATP hydrolysis (Cingolani and Duncan, 2011; Gu et al., 2019; Sobti et al., 2019). Inhibition of ATP hydrolysis appears to be achieved by subunit ζ in *Paraccocus denitrificans* (Morales-Rios et al., 2015; García-Trejo et al., 2016; Varghese et al., 2018), while in mycobacteria extensions from the α subunits interact with the γ subunits to block rotation and ATP hydrolysis (Guo et al., 2021).

While F-type ATP synthases are found in all eukaryotes and are the most common type of ATP synthase in eubacteria, there exist related proton-driven rotary ATP synthases that more closely resemble eukaryotic V-ATPases. Found in archaea and a few eubacteria, these enzymes are known either as prokaryotic V-ATPases, A-ATPases, or in our preferred nomenclature, V/A-ATPases. V/A-ATPases resemble eukaryotic proton-pumping V-type ATPases but have two instead of three peripheral stalks and lack the additional collar subunits found in V-ATPases (Bernal and Stock, 2004; Lau and Rubinstein, 2010; Muench et al., 2011). Further, like F-type ATP synthases, these complexes do not appear to be regulated by the reversible dissociation mechanism that controls V-ATPase activity (Kane, 1995; Sumner et al., 1995). V/A-ATPases can function as ATP synthases, driven by protons or other ions, or as ion pumps (Muench et al., 2011). V/A-ATPases have been subjected to extensive structural analysis by x-ray crystallography, negative stain EM, and cryoEM (Boekema et al., 1999; Bernal and Stock, 2004; Murata et al., 2005; Numoto et al., 2009; Lau and Rubinstein, 2010, 2012; Lee et al., 2010). Similar to F-type ATP synthase, cryoEM has allowed computational separation of rotational states and elucidation of the a subunit fold by evolutionary covariance analysis (Schep et al., 2016), with subsequent high-resolution structures revealing further details of the catalytic mechanism (Zhou and Sazanov, 2019; Kishikawa et al., 2022).

## CryoEM Guides New Therapies

**Figure 5:**
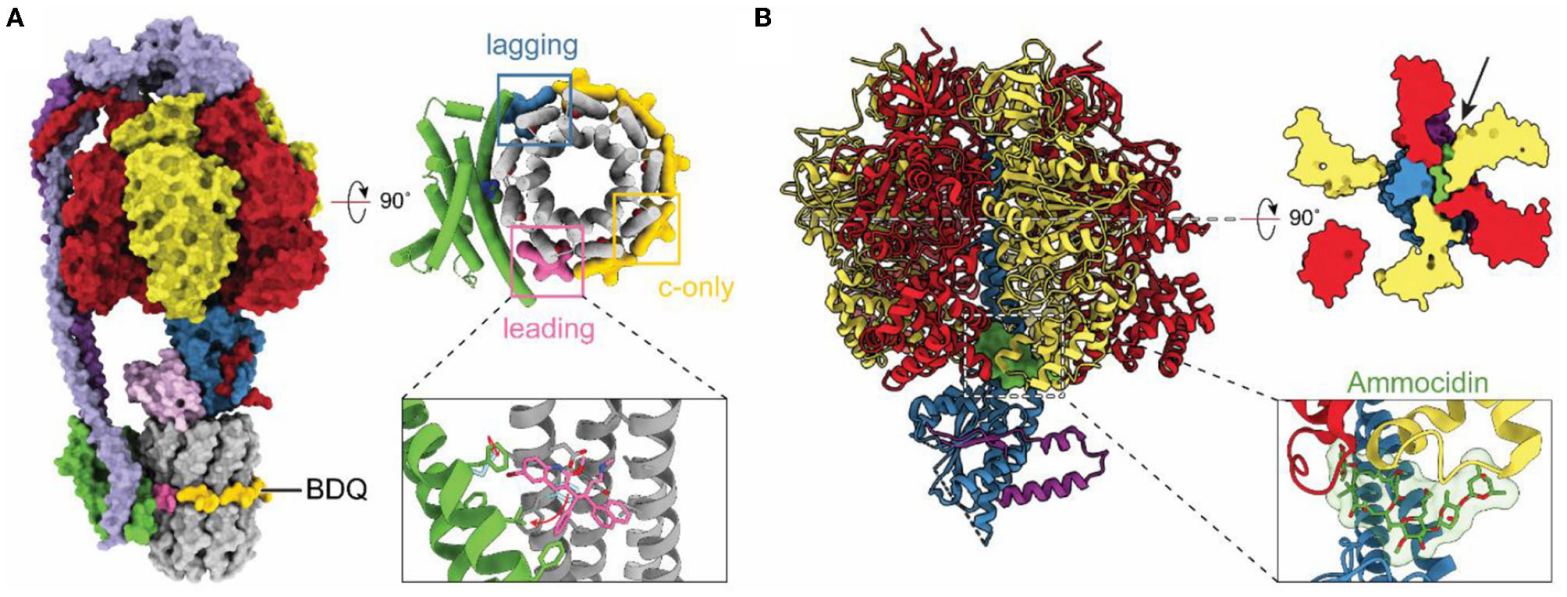
Structures of drug-bound ATP synthases. **(A)** Atomic model of *M. smegmatis* ATP synthase bound to the tuberculosis (TB) drug bedaquiline (BDQ) (PDB: 7JGC) (Guo et al., 2021). Bedaquiline binds at five c-only sites (yellow), a leading site (pink), and a lagging site (blue) in the F_O_ region of the enzyme. Red arrows indicate the movement of residues upon bedaquiline binding. **(B)** Atomic model of *S. cerevisiae* ATP synthase F_1_ region bound to Ammocidin (PDB: 7MD2) (Reisman et al., 2021). Ammocidin (green) binds at the rotor–stator interface (black arrow).

With resolution in cryoEM rivaling or surpassing x-ray crystallography, cryoEM has become an option for investigating how drug molecules interact with ATP synthases in biomedically important forms of the enzyme. The structure of a mycobacterial ATP synthase (Guo et al., 2021) revealed the binding site and conformational changes induced in the enzyme by the compound bedaquiline (Figure 5A), an antibiotic that has revolutionized the treatment of drug-resistant tuberculosis (TB) (Cohen, 2017). Earlier crystallographic studies had already shown how bedaquiline interacts with the ATP synthase c ring (Preiss et al., 2015), but cryoEM with the intact enzyme revealed numerous contacts between the drug and subunit a that explain the drug's high affinity binding and efficacy as a treatment for TB. The structure of an ATP synthase from another major bacterial pathogen, *Acinetobacter baumannii*, has also been determined (Demmer et al., 2021). *A. baumannii* is part of ESKAPE, a group of bacteria that are a leading cause of nosocomial infections and show alarming levels of drug resistance (Oliveira et al., 2020). As ATP synthases are essential for the growth of *A. baumannii* in rich medium (Wang et al., 2014; Gallagher et al., 2015) these structural studies may aid in the development of new antibiotics. The structures of other ATP synthases from biomedically important pathogens have also been determined. These include *T. gondii*, which causes toxoplasmosis and is a model for the plasmodium species that causes malaria (Mühleip et al., 2021). In *Toxoplasma gondii*, disruption of ATP synthase has been shown to affect parasite viability (Huet et al., 2018). Similarly, ATP synthase in *Plasmodium* spp. has been shown to be essential in the mosquito phase of the parasite, with its disruption blocking malaria transmission (Sturm et al., 2015). Finally, the human ATP synthase itself has been identified as a target for cancer therapies with the realization that the glycomacrolides apoptolidin and ammocidin, which display selective cytotoxicity toward transformed cells, inhibit the enzyme (Reisman et al., 2021). The structure of the yeast ATP synthase bound to ammocidin revealed how the compound binds to the F_1_ region to block enzyme activity (Figure 5B; Reisman et al., 2021).

## Conclusions

It has been 60 years since electron microscopy first detected the structures within mitochondrial membranes that would come to be known as ATP synthase. In that time, the capabilities of electron microscopy have expanded from low-resolution imaging of cell ultrastructure to the determination of atomic-resolution structures of macromolecular assemblies such as ATP synthase. Today, cryoEM is revealing the fundamental mechanisms that all ATP synthases have in common, the diversity of ATP synthases throughout biology, and the insights necessary for developing ATP synthase targeting compounds to be used as therapeutics.

## Author Contributions

JR and GC wrote the manuscript and prepared figures. Both authors contributed to the article and approved the submitted version.

## Funding

This study was supported by the Ontario Graduate Scholarship (GC) and Canada Research Chairs (JR).

## Conflict of Interest

The authors declare that the research was conducted in the absence of any commercial or financial relationships that could be construed as a potential conflict of interest.

## Publisher's Note

All claims expressed in this article are solely those of the authors and do not necessarily represent those of their affiliated organizations, or those of the publisher, the editors and the reviewers. Any product that may be evaluated in this article, or claim that may be made by its manufacturer, is not guaranteed or endorsed by the publisher.
